# Safety Pharmacology Study of ET-26 Hydrochloride, a Potential Drug for Intravenous General Anesthesia, in Rats and Beagle Dogs

**DOI:** 10.3389/fphar.2021.679381

**Published:** 2021-05-31

**Authors:** YuJun Zhang, YingYing Jiang, Pan Chang, Yi Kang, DeYing Gong, Jin Liu, WenSheng Zhang

**Affiliations:** ^1^ Department of Anesthesiology, West China Hospital, Sichuan University, Chengdu, China; ^2^ Laboratory of Anesthesia and Critical Care Medicine, Translational Neuroscience Center, West China Hospital, Sichuan University, Chengdu, China; ^3^ National-Local Joint Engineering Research Center of Translational Medicine of Anesthesiology, West China Hospital, Sichuan University, Chengdu, China

**Keywords:** preclinical study, safety pharmacology, intravenous anesthetics, etomidate analogue, ET-26 hydrochloride

## Abstract

**Background:** ET-26 hydrochloride (ET-26HCl), a class 1 new drug, was developed to reserve the advantages of etomidate with a mild adrenocortical inhibition.

**Purpose:** this study was to evaluate the potential adverse effects on the cardiovascular system of beagle dogs and the respiratory and central nervous systems of rats.

**Methods:** three established methods, the whole-body plethysmography for respiratory function, the prototype telemetry transmitter for cardiovascular function, and the standardized functional observational battery for central nervous system function, were accomplished with Good Laboratory Practice standards.

**Results:** no significant difference in the tidal volume, but the respiratory rate and minute ventilation were reduced. The degree of inhibition was the most serious in the first 15 min after dosing and function fully recovered after 1 h. For male rats, the respiratory rate of male rats was reduced significantly at 15 min after injection with ET-26HCl (4 mg/kg, 28.6%, *p* ≤ 0.01; 8 mg/kg, 24.5%, *p* ≤ 0.01; 16 mg/kg, 44.5%, *p* ≤ 0.001), and the minute ventilation at 15 min was decreased by 20.1% (4 mg/kg, *p* = 0.034), 22.2% (8 mg/kg, *p* = 0.019), and 44.6% (16 mg/kg, *p* ≤ 0.001) as compared to control group. As with male rats, the respiratory rate of the female rats was reduced significantly at 15 min (4 mg/kg, 23.3%, *p* ≤ 0.01; 8 mg/kg, 29.2%, *p* ≤ 0.001; 16 mg/kg, 44.1%, *p* ≤ 0.001), and the minute ventilation was decreased by 25.2% (4 mg/kg, *p* ≤ 0.001), 23.0% (8 mg/kg, *p* ≤ 0.01), and 47.6% (16 mg/kg, *p* ≤ 0.001). Then, all the variations in cardiovascular functions were within the expected range for normal biological variation, we concluded that ET-26HCl, even at 10-fold ED_50_, still does not exert toxicological effects on the cardiovascular system. For male beagle dogs, the systolic blood pressure after 24 h following administration of vehicle control or 8, 12, or 16 mg/kg ET-26HCl was 137.80 ± 5.55, 131.76 ± 10.03, 139.88 ± 8.35, and 141.28 ± 8.75 mmHg, respectively. The diastolic blood pressure was 71.16 ± 4.84, 66.52 ± 8.50, 73.64 ± 8.51, and 74.24 ± 8.68 mmHg, respectively. For female beagle dogs, the systolic blood pressure after 24 h following administration of vehicle control or 8, 12, or 16 mg/kg ET-26HCl was 128.28 ± 5.22, 124.76 ± 7.29, 134.88 ± 5.56, and 135.36 ± 8.72 mmHg, respectively. The diastolic blood pressure was 67.00 ± 4.10, 62.12 ± 7.87, 69.44 ± 6.40, and 70.20 ± 8.42 mmHg, respectively. In central nervous system function experiment, all the changes observed in the functional observational battery tests, including motor activity, behavior, coordination, and sensory and motor reflex responses, and reduced body temperature, were resulted in general anesthesia effect of ET-26HCl.

**Conclusion:** ET-26HCl exerts mild, reversible effects on respiratory, cardiovascular, and central nervous system function as verified by standard *in vivo* animal models.

## Introduction

For therapeutic index and cardiovascular stability, etomidate was an ideal drug for intravenous general anesthesia, especially for critically ill or elderly patients (Giese et al., 1985; Tomlin et al., 1998). However, the survival rate of critically ill patients was reduced by the inhibition effect on adrenal cortical hormone of etomidate (Fellows et al., 1983; Kamp and Kress, 2007; Forman, 2011; Chan et al., 2012). But etomidate is still applied in critically ill and elderly patients for anesthesia induction. ET-26 hydrochloride (ET-26HCl), a class 1 new drug approved by the China National Medical Products Administration (NMPA) for clinical trials, was developed to reserve the advantages of etomidate with a mild adrenocortical inhibition.

Compared with etomidate, ET-26HCl had a similar onset time, duration, and recovery time in rats and beagle dogs after single intravenous injection, and it produced a milder effect on adrenocortical function (Wang et al., 2017c; Yang et al., 2017). ET-26HCl also showed similar cardiovascular stability to etomidate in the *ex-vitro* study (Liu et al., 2018) and the *in vivo* studies (Wang et al., 2017a; Wang et al., 2017c; Liu et al., 2018). Due to a higher survival rate, a fewer corticosterone suppression, a lower inflammatory reaction, and a lesser organ injury in the rat lipopolysaccharide-induced sepsis model, ET-26HCl could possibly be safer than etomidate in critically ill patients (Wang et al., 2017b). In the preclinical single- and repeated-dose toxicity studies (Zhang et al., 2020a; Zhang et al., 2020b), ET-26HCl caused slight changes in behavior, laboratory tests findings, organ coefficient, and macroscopic and histopathological examinations. On the basis of observations, ET-26HCl could produce a certainly and safely sedative effect with a mild adrenocortical suppression, which increased the probability of ET-26HCl for clinical use.

The objective of this safety pharmacology study was to evaluate the potential adverse effects on the respiratory, cardiovascular, and central nervous systems by three established methods in accordance with rigorous Good Laboratory Practice (GLP) standards published by the China NMPA: the whole-body plethysmography for respiratory function, the prototype telemetry transmitter for cardiovascular function, and the standardized functional observational battery for central nervous system function.

## Materials and Methods

### Materials

ET-26HCl was synthesized at Yichang Humanwell Pharmaceutical Co., Ltd (China). Propylene glycol was acquired from Spectrum Chemical Manufacturing Co., Ltd (Shanghai, China). Sterile injection water was purchased from Suzhou Jiushuntang Pharmacy Co., Ltd (China). Propylene glycol (35% *v/v*) was diluted using sterile injection water and was used to prepare the ET-26HCl solution. All the solutions were filtered through a 0.22 μm syringe filter, stored at 2–8°C, and used within 10 days.

### Animals

Adult Sprague-Dawley rats (aged 7–9 weeks, weighing of male and female rats for respiratory function study, 191.58–230.07 g and 176.21–206.00 g; for central nervous system function study, 205.30–245.67 g and 181.67–210.06 g) were purchased from Beijing Vital River Laboratory Animal Technology Co., Ltd (China). Beagle dogs (1–2 years, 6.3–15 kg body weight) were purchased from Beijing Marshall Biotechnology Co., Ltd (China). Animals were maintained in a controlled environment with a 12 h light/dark cycle. Food and water were provided *ad libitum*. All animals underwent a minimum of a 5 days adaptation period in the animal facility before the initiation of the tests. All experimental procedures were approved by the Institutional Animal Care and Use Committee of West China Hospital, Sichuan University, Chengdu, China (2015015A).

### Core Battery Experimental Design

The experimental methods for the core battery tests, including effects on the respiratory, cardiovascular, and central nervous systems, was drafted based on the Guideline on Safety Pharmacology Studies for Human Pharmaceuticals by the International Council for Harmonization of Technical Requirements for Pharmaceuticals for Human Use (ICH) Harmonized Tripartite Guideline S7A (Guideline, 2001) and the Guideline on Safety Pharmacology by the CFDA published in 2014 (Guideline, 2014). Dosage chosen were 4, 8, 16 mg/kg for rat and 8, 12, 16 mg/kg for beagle dog, approximating or exceeding the 2-fold median effective dose of ET-26HCl in rats (4.2 mg/kg) and beagle dogs (2.1 mg/kg).

### Respiratory Function

Forty rats (*n* = 10 in each group, five male and five female) were divided into four randomized groups to investigate the effects of ET-26HCl on respiratory function. ET-26HCl (10 mg/ml) at three doses (4 mg/kg, 0.4 ml/kg; 8 mg/kg, 0.8 ml/kg; and 16 mg/kg, 1.6 ml/kg) and vehicle control (35% *v/v* propylene glycol, 1.6 ml/kg) were injected separately through the vena caudalis at a rate of 0.04 ml/s according to the method described in previous study (Hoymann, 2007). The rats were put into the Head-Out plethysmographs chamber (Data Sciences International [DSI], St. Paul, MN, United States ) for at least 5 min for adaption, and their tidal volume, respiratory rate, and minute ventilation were recorded as baseline. The rats were then injected and their characteristics were collected at the following time points: 15 min, 30 min, 1, 2, 4, and 24 h. The collected data were recorded and analyzed using the Ponemah P3 Plus software (version 5.20, DSI). Respiratory parameters were recorded as an average of a 5 s period, and the data of each time point are presented as an average of the data over 15 min.

### Cardiovascular Function

Six beagle dogs (*n* = 6 in each group, three male and three female) were used to investigate the effects of ET-26HCl on cardiovascular function. ET-26HCl (10 mg/ml) at three doses (8 mg/kg, 0.8 ml/kg; 12 mg/kg, 1.2 ml/kg; and 16 mg/kg, 1.6 ml/kg) and vehicle control were injected separately through the vein at 40 ± 4 s. In each group, the beagle dogs were given at least 7 days for wash-out. Before administering the drugs, all beagle dogs were fasted overnight (8 h for food and 4 ± 0.5 h for water), and food and water were supplied post dosing.

Surgical instrumentation was conducted according to published studies (Chui et al., 2009; Kearney et al., 2010). Briefly, the beagle dogs were subjected to effective sedation and pain control, and all surgical procedures were performed under standard aseptic techniques. The prototype telemetry transmitter (TL11M2-D70-PCT, DSI) was implanted intramuscularly at approximately the level of the third lumbar vertebra, between external and internal abdominal oblique muscles. A blood pressure catheter was placed in the right femoral artery. Meanwhile, electrocardiographic (ECG) leads were placed in an approximate lead II configuration, with the negative lead routed subcutaneously in the right axillary area below the pectoral muscles against the first interspace, and the positive lead placed in an intercostal space on the left caudo-lateral thorax at the 9–10 rib space. After surgery, the transmitter was used to record ECG, blood pressure, and heart rate. All parameters were measured and analyzed using Ponemah P3 Plus software (version 5.20, DSI).

For at least 2 h prior to administration of each dose, continuous recordings of arterial pressure and ECG were collected to ensure normal cardiovascular parameters and functional transmitters using a 10 s logging rate (baseline). Blood pressure and ECG were recorded and all derived parameters were logged as means of 1 min periods to approximately 24 h after dosing. Reported time points were taken as the average of periods of 1 h except pre-dose, which was the average of a single period of 2 h. ECG tracings of a minimum 30 s duration were obtained from all the dogs twice (at least 30 min apart) prior to each dose and at 6 post-dose time points (0–0.5 h, 0.5–1 h, 1–1.5 h, 4–4.5 h, 8–8.5 h, and 24 h). The raw ECG data were evaluated by a certified veterinary cardiologist for waveform abnormalities and arrhythmias.

### Central Nervous System Function

Forty rats (*n* = 10 in each group, five male and five female) were divided into four randomized groups to investigate the effects of ET-26HCl on central nervous system function. ET-26HCl at three doses (4, 8, and 16 mg/kg) and vehicle control were injected separately through the vena caudalis at a rate of 0.04 ml/s. A standardized functional observational battery (FOB) was used to determine the central nervous system safety profile of ET-26HCl by measuring general motor activity, behavioral changes, coordination, sensory and motor reflex responses, and body temperatures (Moscardo et al., 2007; Gauvin et al., 2016). The FOB observation targets were assessed at 0 min, 5 min, 1.5 h, and 24 h and the following parameters were recorded: 1) cage observations: posture, tremors, convulsions, abnormal movements, reactivity to removal from cage; 2) open-field observations: vertical movement, defecation, urination, gait, tremors, convulsions, abnormal movements/behavior, piloerection, grooming; 3) hand-held observations: olfactory response, responsiveness to tail pinch, touch, and noise, salivation, muscle tone, extensor-thrust response, respiration, eyelid reflex, pupil size; 4) measurements: rectal temperature, forelimb and hindlimb grip force, landing foot splay length. All behavior analysis and measurements were performed by trained professional investigators, and any behavioral changes or clinical symptoms of toxicity were recorded.

### Statistical Analysis

All data from male and female animals were analyzed separately to distinguish the potential adverse effects based on sex. For respiratory function central nervous system function, the homogeneity-variance data were assessed by one-way analysis of variance (ANOVA) followed by Dunnett’s test to compare the treatment groups with the control. Otherwise, the data were assessed by Kruskal-Wallis test followed by Dunnett’s test to compare with the control. Repeated measurements were assessed by repeated measures ANOVA, and the differences between the treatment groups and the control group were assessed by *t*-test of least squares, followed by Bonferroni correction to avoid type I errors. All qualitative data were assessed by Fisher’s exact test or Chi-square test. For cardiovascular function, the data were assessed by analysis of variance for repeated measurements with the covariance analysis model of compound symmetry and heterogeneous compound symmetry. The level of statistical significance was set at *p* < 0.05. The data were analyzed using SAS (version 9.1.3, SAS Institute, Cary, NC, United States).

## Results

### Respiratory Function Changes

**FIGURE 1:**
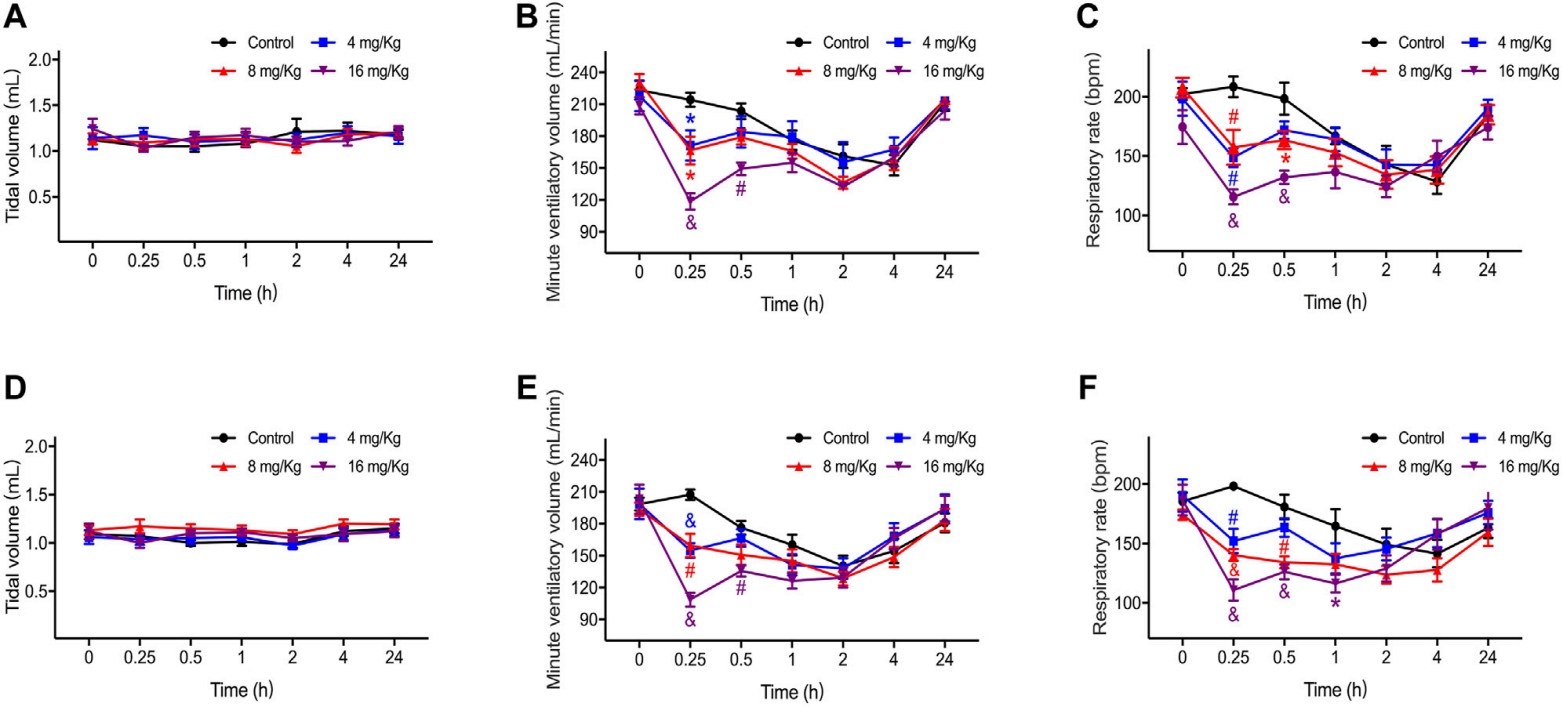
The result of tidal volume, respiratory rate and minute ventilation in male **(A–C)** and female **(D–F)** rats. Data were depicted as the mean ± SEM. Compared with control group, *p < 0.05; #*p* ≤ 0.01; & *p* ≤ 0.001.

The tidal volume, respiratory rate, and minute ventilation of rats are presented in Figure 1 (A-C for male and D-F for female). There was no significant difference in the tidal volume (Figures 1A,D) between the rats in all four groups, but the respiratory rate and minute ventilation were reduced after ET-26HCl administration. The degree of inhibition was the most serious in the first 15 min after dosing and function fully recovered after 1 h (Figures 1B,C). In male rats treated with ET-26HCl (Figure 1B), minute ventilation at 15 min was decreased by 20.1% (4 mg/kg, *p* = 0.034), 22.2% (8 mg/kg, *p* = 0.019), and 44.6% (16 mg/kg, *p* ≤ 0.001) as compared to that in rats of the control group. However, only those rats treated with 16 mg/kg ET-26HCl did not recover their minute ventilation by 30 min (−26.7%, *p* ≤ 0.01). The respiratory rate of male rats (Figure 1C) was reduced significantly at 15 min after injection with ET-26HCl at 4 mg/kg (28.6%, *p* ≤ 0.01), 8 mg/kg (24.5%, *p* ≤ 0.01), and 16 mg/kg (44.5%, *p* ≤ 0.001). The inhibitory effect of ET-26HCl on the respiratory rate at 8 and 16 mg/kg was persisted for 30 min (−17.7%, *p* = 0.037; −33.4%, *p* ≤ 0.001, respectively).

As with male rats, the minute ventilation of female rats (Figure 1E) at 15 min was decreased by 25.2% (4 mg/kg, *p* ≤ 0.001), 23.0% (8 mg/kg, *p* ≤ 0.01), and 47.6% (16 mg/kg, *p* ≤ 0.001), and only rats treated with 16 mg/kg ET-26HCl showed a significant difference as compared to those in the control group at 30 min (−23.0%, *p* ≤ 0.01). The respiratory rate (Figure 1F) of the female rats administered with ET-26HCl at 15 min was reduced by 23.3% (4 mg/kg, *p* ≤ 0.01), 29.2% (8 mg/kg, *p* ≤ 0.001), and 44.1% (16 mg/kg, *p* ≤ 0.001). After dosing at 30 min, ET-26HCl reduced the respiratory rate at doses of 8 and 16 mg/kg (25.8%, *p* ≤ 0.01; 30.2%, *p* ≤ 0.001, respectively). Even at 1 h, the female rats in the 16 mg/kg group had a slower respiratory rate as compared to that of the control rats (−29.3%, *p* = 0.02).

### Cardiovascular Function Changes

**FIGURE 2:**
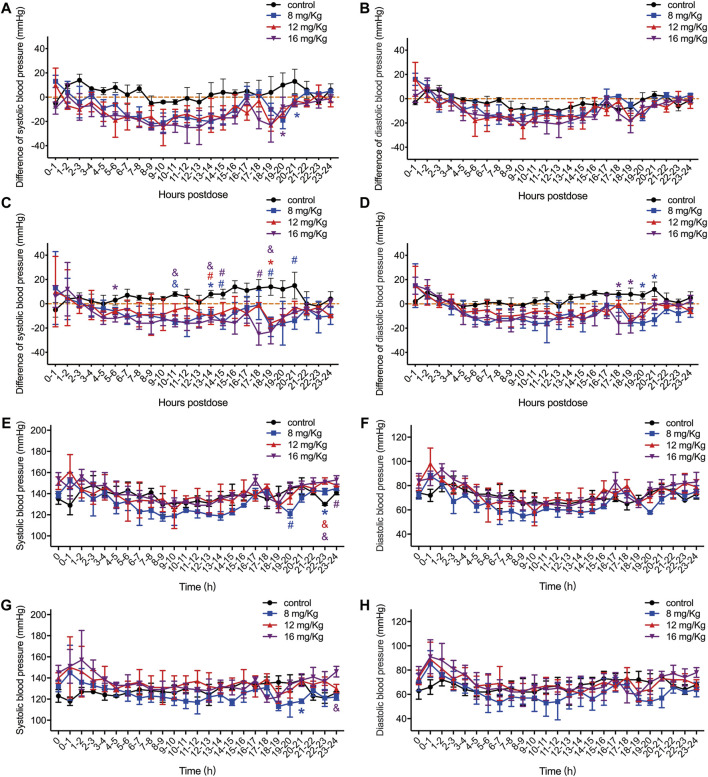
The difference value of systolic blood pressure, diastolic blood pressure and QRS interval in male **(A–B)** and female **(C–D)** rats, and the original value of systolic blood pressure and diastolic blood pressure in male **(E–F)** and female **(G–H)** rats. The difference value was calculated from the measured value minus the mean value for the two hours before drug administration. Data were depicted as the mean ± SD. Compared with control group, *p < 0.05; #*p* ≤ 0.01; & *p* ≤ 0.001.

**FIGURE 3:**
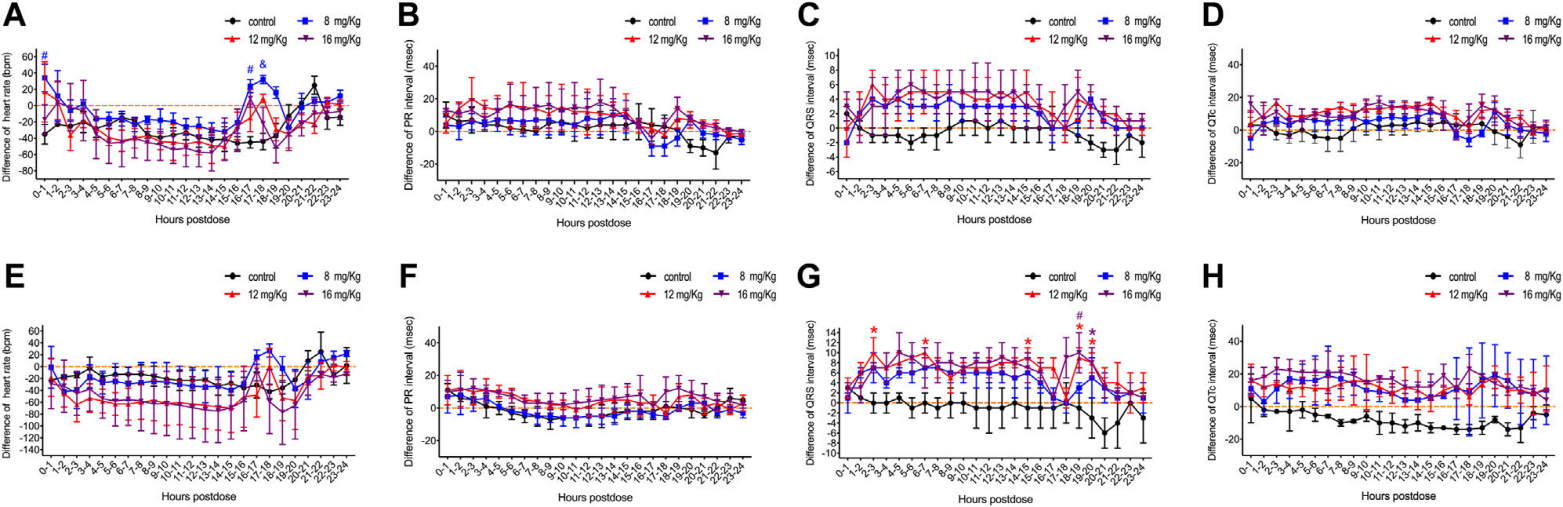
The difference value of heart rate, QRS interval, PR interval and QT interval in male **(A–C)** and female **(D–F)** rats. The difference value was calculated from the measured value minus the mean value for the two hours before drug administration. Data were depicted as the mean ± SD. Compared with control group, *p < 0.05; #*p* ≤ 0.01; & *p* ≤ 0.001.

When evaluating the safety of ET-26HCl, its minimal effect on the cardiovascular system was the most important driver for further drug development. In this study, we employed a telemetry device to measure cardiovascular parameters in sober beagle dogs. The results of blood pressure and ECG analysis are presented in Figures 2, 3.

After administration of ET-26HCl at doses of 8, 12, and 16 mg/kg, the differences in both systolic and diastolic blood pressure were reduced in male and female beagle dogs (Figure 2). The average maximum difference in systolic blood pressure decreased at 25 mmHg for male and female beagle dogs (Figures 2A,C). The maximum average difference in diastolic blood pressure in male and female beagle dogs reduced at 23 and 16 mmHg, respectively (Figures 2B,D). In male beagle dogs (Figures 2E,F), the systolic blood pressure after 24 h following administration of vehicle control or 8, 12, or 16 mg/kg ET-26HCl was 137.80 ± 5.55, 131.76 ± 10.03, 139.88 ± 8.35, and 141.28 ± 8.75 mmHg, respectively. The diastolic blood pressure was 71.16 ± 4.84, 66.52 ± 8.50, 73.64 ± 8.51, and 74.24 ± 8.68 mmHg, respectively. In female beagle dogs (Figures 2G,H), the systolic blood pressure after 24 h following administration of vehicle control or 8, 12, or 16 mg/kg ET-26HCl was 128.28 ± 5.22, 124.76 ± 7.29, 134.88 ± 5.56, and 135.36 ± 8.72 mmHg, respectively. The diastolic blood pressure was 67.00 ± 4.10, 62.12 ± 7.87, 69.44 ± 6.40, and 70.20 ± 8.42 mmHg, respectively. However, the systolic blood pressure decreased significantly at a few (3 out of 24) time points (Figures 2A,C). All reduced blood pressure values were within the normal biological range.

Concurrently, ECG was obtained for a more comprehensive evaluation of cardiovascular system function. ET-26HCl had no significant influence on the heart rate and the PR interval of male (Figures 3A,B) or female (Figures 3E,F) beagle dogs. Overall, the beagle dogs injected with ET-26HCl had an increased difference in QRS intervals. However, when separated by sex, males showed no significant difference as compared to the control (Figure 3C), but ET-26HCl administration significantly increased the difference in QRS intervals in female beagle dogs at 12 mg/kg over the 2–3 h, 6–7 h, 14–15 h, and 18–20 h intervals, and at 16 mg/kg over the 18–20 h interval (Figure 3G). The maximally prolonged QTc interval (Figures 3D,H) was observed at 10–11 h for males (17 ms, +6.8%) and at 2–3 h and 17–18 h for females (23 ms, +9.4%). However, these changes were not significantly different when compared to the control.

As all the variations in cardiovascular functions were within the expected range for normal biological variation, we concluded that ET-26HCl, even at 10-fold ED_50_, still does not exert toxicological effects on the cardiovascular system.

### Central Nervous System Function Changes

A standardized FOB experiment was performed to determine the central nervous system safety profile of ET-26HCl by estimating the motor activity, behavioral changes, coordination, sensory and motor reflex responses, and body temperatures. Following administration of ET-26HCl, hematuria was observed in 40% of male and 100% of female rats in the 4 mg/kg group, 80% of male and 80% of female rats in the 8 mg/kg group, and 20% of male and 80% of female rats in the 16 mg/kg group. Nevertheless, hematuria was also observed in all the rats in control group.

**FIGURE 4:**
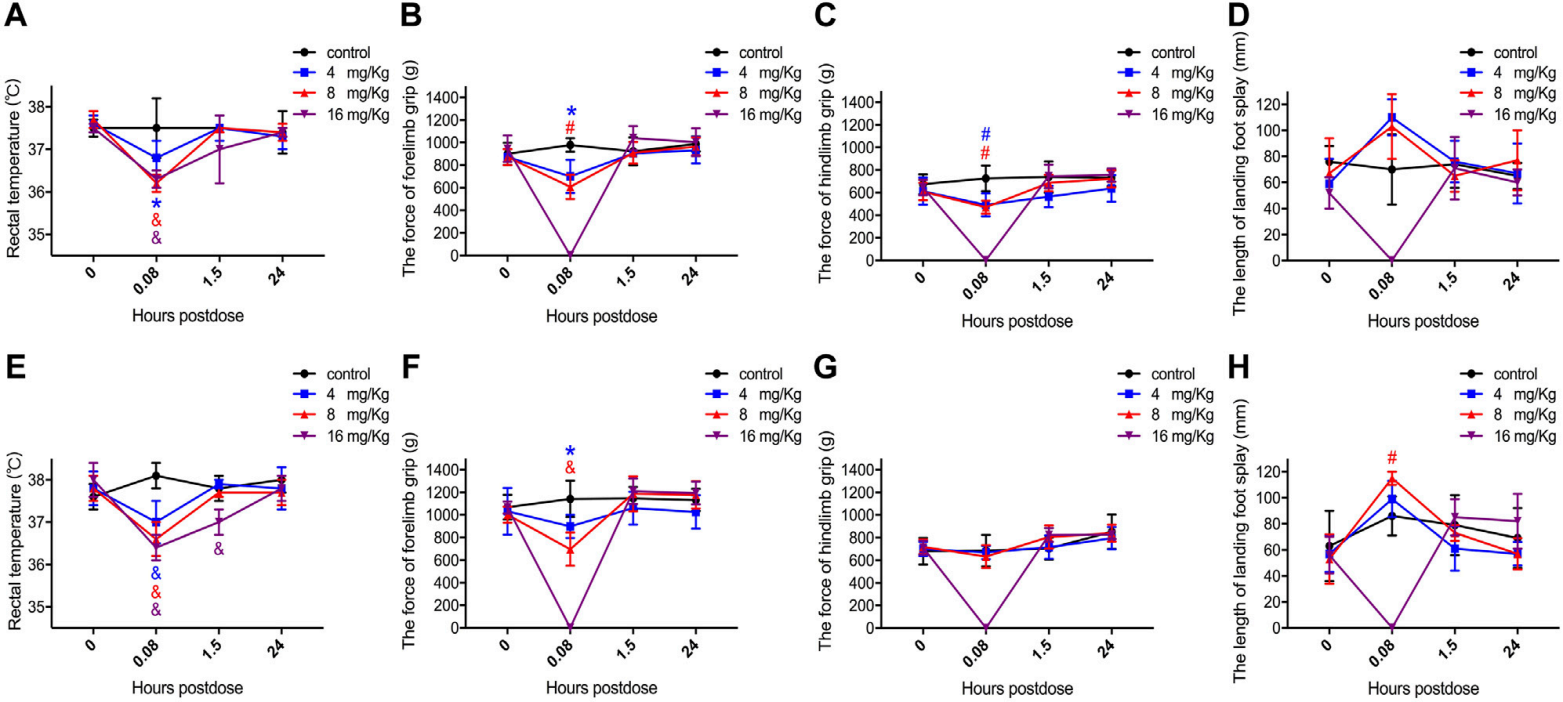
The value of rectal temperature, forelimb grip force, hindlimb grip force and landing foot splay length in male **(A–D)** and female **(E–H)** rats. Data were depicted as the mean ± SD. Compared with control group, *p < 0.05; #*p* ≤ 0.01; & *p* ≤ 0.001.

Changes in the central nervous system functions were clearly observed within the first 5 min after injection of ET-26HCl at all doses. The symptoms are detailed in Table 1; the rats demonstrated abnormal posture, decrease in locomotor activity, abnormal gait, lack of grooming, lack of responsiveness to touch, lack of olfactory response, lack of eyelid reflex, hypopnea, decrease in muscle tone, etc. Moreover, we estimated the rectal temperature, forelimb and hindlimb grip force, and length of landing foot splay (Figure 4). Only rectal temperature was measured in rats in the 16 mg/kg group at 5 min due to their general anesthetized status. ET-26HCl resulted in decreased rectal temperature in male rats (36.8 ± 0.4°C at 4 mg/kg, *p* = 0.028; 36.2 ± 0.2°C at 8 mg/kg, *p* ≤ 0.001; 36.3 ± 0.2°C at 16 mg/kg, *p* ≤ 0.001; Figure 4A) and in female rats (37.0 ± 0.5°C at 4 mg/kg, *p* ≤ 0.001; 36.6 ± 0.4°C at 8 mg/kg, *p* ≤ 0.001; 36.4 ± 0.3°C at 8 mg/kg, *p* ≤ 0.001; Figure 4D). However, rectal temperature returned to normal by the 1.5 h time point. Additionally, the forelimb grip force of male rats was significantly decreased 5 min after treatment with ET-26HCl (Figure 4B) in the 4 and 8 mg/kg groups (700.4 ± 146.8 g, *p* = 0.015; and 608 ± 106.7 g, *p* ≤ 0.01, respectively) as compared to that in the control group (978.1 ± 60.5 g). The forelimb grip force in the female rats (Figure 4E) was 898.8 ± 102.0 g in the 4 mg/kg group (*p* = 0.019), 679.4 ± 147.5 g in the 8 mg/kg group (*p* ≤ 0.001), and 1,142.3 ± 161.5 g in the control group. As compared to the control group (725.2 ± 113.4 g), hindlimb grip force of male rats (Figure 4C) was reduced to 491.8 ± 101.4 g in the 4 mg/kg group (*p* ≤ 0.01) and 470.9 ± 57.5 g in the 8 mg/kg group (*p* ≤ 0.01). The length of landing foot splay of female rats was increased to 115 ± 5 mm in the 8 mg/kg group (*p* ≤ 0.01, Figure 4H). In the FOB tests, all the observed changes followed a dose-effect relationship regardless of gender, and most of the values returned to normal levels within 1.5 h. ET-26HCl-induced status of general anesthesia resulted in changes in motor activity, behavior, coordination, and sensory and motor reflex responses, and body temperature.

**TABLE 1:** The result of functional observational battery experiments (*n* = 10).

FOB parameters	Gender	5 min				1.5 h			
		Control	4 mg/kg	8 mg/kg	16 mg/kg	Control	4 mg/kg	8 mg/kg	16 mg/kg
Abnormal posture (curl up, crouch or lie on the side)	Male	—	5/5	5/5	5/5	—	—	—	—
	Female	1/5	2/5	5/5	5/5	—	—	—	—
Mild tremor (cage observation)	Male	—	2/5	4/5	2/5	—	—	—	—
	Female	—	—	5/5	1/5	—	—	—	—
Vertical movement (mean value)	Male	4	0	0	0	3	2	4	2
	Female	6	0	0	0	7	7	6	3
No locomotor activity	Male	—	—	4/5	5/5	—	—	—	—
	Female	—	—	5/5	5/5	—	—	—	—
Low alertness	Male	—	3/5	5/5	5/5	1/5	1/5	—	—
	Female	—	2/5	5/5	5/5	—	—	—	—
Abnormal gait	Male	—	5/5	5/5	5/5	—	—	—	—
	Female	—	5/5	5/5	5/5	—	—	—	—
Mild tremor (open-field observation)	Male	—	—	4/5	2/5	—	—	—	—
	Female	—	—	4/5	2/5	—	—	—	—
No grooming	Male	3/5	4/5	5/5	5/5	2/5	4/5	2/5	4/5
	Female	3/5	5/5	5/5	5/5	3/5	5/5	3/5	4/5
No responsiveness to touch	Male	—	1/5	3/5	5/5	—	—	1/5	1/5
	Female	1/5	1/5	5/5	5/5	—	—	2/5	—
No olfactory response	Male	—	—	—	5/5	—	—	—	—
	Female	—	—	—	5/5	—	1/5	—	—
Hypopnea	Male	—	1/5	2/5	5/5	—	—	—	—
	Female	—	—	—	5/5	—	—	—	—
1/2 eyelid close	Male	—	—	—	4/5	—	—	—	—
	Female	—	—	—	5/5	—	—	—	—
No eyelid reflex	Male	—	—	1/5	5/5	—	—	—	—
	Female	—	—	—	5/5	—	—	—	—
Muscle tone decreases	Male	—	2/5	4/5	5/5	—	—	—	—
	Female	—	—	5/5	5/5	—	—	—	—
Miosis	Male	—	1/5	3/5	5/5	—	—	—	—
	Female	—	2/5	5/5	5/5	—	—	—	—
No light reflex	Male	—	—	—	5/5	—	—	—	—
	Female	—	—	—	5/5	—	—	—	—

## Discussion

The major purpose of this study is to predict the potential adverse effects by the standard *in vivo* animal models in rats and beagle dogs, and ET-26HCl produced mild and reversible influences on respiratory, cardiovascular, and central nervous system functions.

The advantages of etomidate have hemodynamic stability, minimal respiratory depression after either a single or continuous administration. For respiratory system, induction with etomidate produces a slight increase in PaCO_2_ (±15%) with no change in PaO_2_ which was induced by a transient hyperventilation followed by a similarly brief period of apnea (Morgan et al., 1977; Colvin et al., 1979; Craig et al., 1982). As for the effects of ET-26HCl on the respiratory system, breathing rate and minute ventilation decreased slightly within 15 min after injection. According to the result from up-and-down study, the ED_50_ of ET-26HCl in beagle dogs was 1.44 mg/kg, and the mean time from disappearance of the righting reflex to free walking was 5.05 min after administered with 2-fold ED_50_ (Yang et al., 2017). The doses of ET-26HCl in this preclinical safety pharmacology study were 6, 8 and 11-fold ED_50_. Meanwhile, the pharmacokinetic features of ET-26HCl had a linear relationship in beagle dogs (Zhang et al., 2019). Thus, the duration of general anesthesia might reach to 15, 20 and 25 min after administered at 8, 12, and 16 ml/kg ET-26HCl, respectively. The main change of respiratory system was anesthetic sedation-induced respiratory rate slowness, and then the minute ventilation was decreased. However, no apnea was observed after dosing and the function was fully recovered by self without any first-aid treatments. All the changes were the results of the general anesthetic effects of ET-26HCl, and were within the expected ranges of normal biological changes. Thus, these were not toxicologically significant.

For cardiovascular system, etomidate administered with induction dose (0.3 mg/kg) produces almost no change in heart rate, mean arterial pressure, mean pulmonary artery pressure, pulmonary capillary wedge pressure, central venous pressure, stroke volume, cardiac index, and pulmonary and systemic vascular resistance (Gooding et al., 1979). Even dose increased with 50% (0.45 mg/kg), etomidate also produces minimal changes in cardiovascular variables (Criado et al., 1980). To assess the functions of the cardiovascular system, we used a similar telemetry method to determine the mean arterial pressure of rats as reported in our previous study (Wang et al., 2017c). Administration of ET-26HCl produced a slight reduction in blood pressure with a lower maximum effect and a shorter duration than both etomidate and propofol at equal hypnotic dosage. Furthermore, ET-26HCl resulted in similarly stable hemodynamics when compared with etomidate in rats with uncontrolled hemorrhagic shock (Wang et al., 2017a). In beagle dogs, ET-26HCl, at doses of between 6- and 12-fold ED_50_ mildly decreased blood pressure and lengthened the QRS and QTc intervals. Given that these changes were a result of the general anesthetic effect, ET-26HCl was found to be similar to etomidate in terms of hemodynamic stability (Wang et al., 2017a; Wang et al., 2017b; Wang et al., 2017c; Liu et al., 2018), and therefore has the potential to be a novel general anesthetic for use in elderly and critically ill patients.

The main limitation of etomidate was to decrease the survival rate of critically ill by its inhibition effect on adrenal cortical hormone (Fellows et al., 1983; Kamp and Kress, 2007; Forman, 2011; Chan et al., 2012). ET-26HCl was developed to reserve the good cardiovascular stability of etomidate with a mild adrenocortical inhibition. In our previous study, we demonstrated that ET-26HCl produced a similar cardiovascular stability to etomidate in the *ex-vitro* study (Liu et al., 2018) and the *in vivo* studies (Wang et al., 2017a; Wang et al., 2017c; Liu et al., 2018). Meanwhile, ET-26HCl produced a milder effect on adrenocortical function than etomidate (Wang et al., 2017c; Yang et al., 2017). In the preclinical single- and repeated-dose toxicity studies (Zhang et al., 2020a; Zhang et al., 2020b), ET-26HCl caused slight changes in behavior, laboratory tests findings, organ coefficient, and macroscopic and histopathological examinations. On the basis of observations, ET-26HCl could produce a certainly and safely sedative effect with a mild adrenocortical suppression, which increased the probability of ET-26HCl for clinical use.

In pre-clinical experiments, ET-26HCl has been proven to retain the advantages of etomidate, only mildly inhibiting the functions of the respiratory, cardiovascular, and central nervous systems. All these findings demonstrate the potential for the clinical use of ET-26HCl and afford guidance for further clinical studies. When designing a phase I clinical trial, attention should be paid to the following: first, urine occult blood tests should be conducted; next, a manual and spontaneous breathing device should be used in case it is needed for preventing apnea; finally, blood pressure and ECG must be continuously monitored.

## Conclusion

Our core battery experiments on pharmacological safety identified potential adverse effects that may occur in further clinical trials evaluating the use of ET-26HCl, a novel etomidate analogue, as a general anesthetic. In this study, we demonstrated that ET-26HCl exerts mild, reversible effects on respiratory, cardiovascular, and central nervous system function as verified by standard *in vivo* animal models, further demonstrating the potential for the clinical use of ET-26HCl.

## Data Availability

The raw data supporting the conclusion of this article will be made available by the authors, without undue reservation.
